# Diagnostic implications of Gardner Syndrome, case report of a familial adenomatous polyposis (FAP) variant, for eye care professionals

**DOI:** 10.1016/j.ijscr.2024.109379

**Published:** 2024-02-10

**Authors:** Adrian Babel, Eric K. Chin, David RP Almeida

**Affiliations:** a Boonshoft School of Medicine, Dayton, OH, USA; b Retina Consultants of Southern California, Redlands, CA, USA; c Loma Linda Eye Institute, Veterans Affair Hospital, Loma Linda, CA, USA; d Erie Retinal Surgery & Erie Retina Research, Erie, PA, USA

**Keywords:** Gardner syndrome (GS), Familial adenomatous polyposis (FAP), Congenital hypertrophy of retinal epithelium (CHRPE)

## Abstract

**Introduction:**

Gardner Syndrome (GS) is a variant of Familial Adenomatous Polyposis (FAP). FAP is characterized by several precancerous adenomatous intestinal polyps while GS has additional distinct extraintestinal features such as congenital hypertrophy of retinal epithelium (CHRPE), which we describe here.

**Presentation of case:**

42-year-old male with GS presenting with flashes and floaters observed to have CHRPE-like lesions characteristic of GS.

**Discussion:**

Subtle CHRPE findings differentiate pathological, described in the present case, from non-pathological etiologies and may guide further management.

**Conclusion:**

Here we present the signs and symptoms that raise suspicion for GS associated with CHRPE and how to approach management late in the disease presentation.

## Highlights

• Gardner Syndrome is a variant of Familial Adenomatous Polyposis.
• Retinal lesions are an early manifestation of Gardner Syndrome.
• Bilateral retinal findings in Gardner Syndrome are specific to this condition.
• Proper identification of retinal lesions may direct subsequent management.

## 1 Introduction

Gardner Syndrome (GS) is a rare variant of Familial Adenomatous Polyposis (FAP) that is characterized by extraintestinal manifestations in addition to the numerous adenomatous intestinal polyps classically observed in FAP [[1], [2], [3], [4], [5]]. Colonic polyps are the prevailing sign in FAP and have a strikingly high propensity for malignant transformation (up to 100 % if left untreated) during the lifetime of patients with FAP [[1], [2], [3], [4]]. Early symptoms that may suggest the presence of colonic polyps and a potential polyposis syndrome include cramping, obstructive constipation, rectal bleeding, diarrhea, and/or vomiting [1]. Once FAP or GS is discovered via various diagnostic modalities or familial inheritance, preventative treatment measures such as NSAIDs or COX2 inhibitors, which decrease colonic polyp growth, are initiated. Yearly colonoscopies (with known polyps) or sigmoidoscopies (no known polyps) starting at 10–15 years of age are indicated for adequate surveillance of this condition. While medical therapies slow polyp growth, if >20 colonic polyps are identified, colonic resection is recommended and is currently the only definitive cure to prevent colorectal cancer development, which would otherwise occur by age 40 [[1], [2], [3], [4]].

FAP and GS are typically inherited in an autosomal-dominant pattern caused by germline mutations within band 5q21, which is linked to the tumor suppressor gene adenomatous polyposis coli (APC) on chromosome 5 [[1], [2], [3], [4], [5]]. It is estimated that 11–33 % of patients with FAP acquire this condition via de novo mutations to the APC gene [[2], [3], [4]]. The four main phenotypic variants resulting from APC mutations are FAP, Turcot Syndrome, GS, and attenuated FAP [1,3,4]. In the United States, the incidence of GS is 1 in 8000 individuals, and the prevalence is one in one million individuals [1,3]. Loss of APC gene function results in uncontrolled cell division and growth which causes the intestinal adenomatous polyps seen in FAP and the extraintestinal manifestations observed in GS [[1], [2], [3], [4], [5]].

Extracolonic manifestations of GS include desmoid tumors, osteomas of the mandible, orbit, and skull, lipomas, fibromas, epidermoid cysts, dental abnormalities, and congenital hypertrophy of the retina pigment epithelium (CHRPE) [[1], [2], [3], [4], [5], [6]]. Ophthalmic identification of CHRPE is commonly the earliest sign of GS in patients with or without a known GS diagnosis and can be observed in early childhood [3,7]. 84 % of CHRPE lesions in FAP are located in the retinal periphery, therefore imaging modalities that evaluate peripheral retina must be utilized to screen for and identify CHRPE lesions adequately [3,8,9]. Such imaging modalities described in the literature include dilated fundoscopic examination via slit lamp and/or indirect ophthalmoscopy, wide-field scanning-laser ophthalmoscopy (recommended screening tool), color fundus photography (effective for follow-up comparisons), fundus autofluorescence, fluorescein angiography, optical coherence tomography (OCT), or OCT angiography [3,8,10,11]. A 2014 retrospective case series suggested that CHRPE can be observed in 90 % of patients with FAP, with a 76 % sensitivity and 92 % specificity for the presence of colonic polyps in FAP [7]. More recently, distinct differences have been identified in CHRPE and CHRPE-like lesions that point toward or away from polyposis syndromes such as GS [[2], [3], [4],^8^]. These distinctions are critical for clinicians to avoid misdiagnosis and overtreatment and to determine appropriate therapies and management of patients with these findings [2,8].

CHRPE may be congenital and benign without associated systemic conditions; therefore, this finding does not always suggest a GS diagnosis [[2], [3], [4],^8^]. These congenital CHRPE lesions have recently been renamed typical CHRPE. They are characterized by solitary, flat, unilateral lesions with smooth borders that are typically (88 %) highly pigmented and occasionally (52 %) have a hypopigmented “halo” surrounding the lesions [8]. The prevalence of typical CHRPEs in the general population is significantly variable, with studies suggesting 0.4 %–30 % prevalence [8]. One percent of typical CHRPE cases have adenocarcinoma transformation, with no reports of metastasis [2]. Another congenital and benign CHRPE-like lesion is called a grouped pigmentation of the retina (also known as “bear tracks”), which is characterized by unilateral multifocal clusters (3–30 lesions) within a single retinal quadrant [8].

The CHRPEs observed in the present case are associated with genetic polyposis syndromes, such as GS, and have been recently renamed as pigmented ocular fundus lesions (PO-FLs) of FAP [8]. These lesions resemble bear tracks but are typically bilateral, occur in multiple retinal quadrants, and have irregular and depigmented borders [2,3,8]. PO-FLs have a characteristic oval “pisciform” (fish-shaped) appearance and typically do not directly threaten vision [2,3,8]. The presence of bilateral CHRPE-like lesions with a presentation consistent with PO-FLs should raise clinical suspicion for an underlying systemic disorder and warrants further diagnostic workup with colonoscopy to screen for polyps and genetic screening if ≥10 polyps are present [4,8]. It is worth noting that while CHRPE-like lesions do not typically cause vision changes and may be asymptomatic, orbital osteomas found in GS may present with vision changes due to their mass effect on surrounding tissues and should strongly suggest GS in the setting of concurrent PO-FLs [6]. Furthermore, CHRPE absence does not exclude GS [[2], [3], [4]]. Here, we report a case of bilateral PO-FLs in a patient with a history of GS.

## 2 Observation

**Fig. 1:**
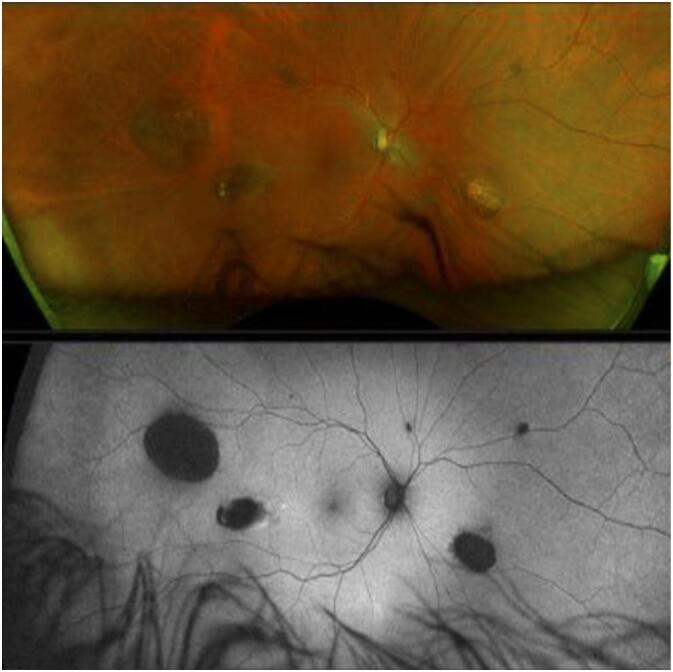
Initial fundoscopic examination OD revealed small to large round CHRPE-like lesions without holes, tears, or macular involvement in multiple retinal quadrants consistent with PO-FLs (oval, fish-tail depigmentation at lesion margin).

**Fig. 2:**
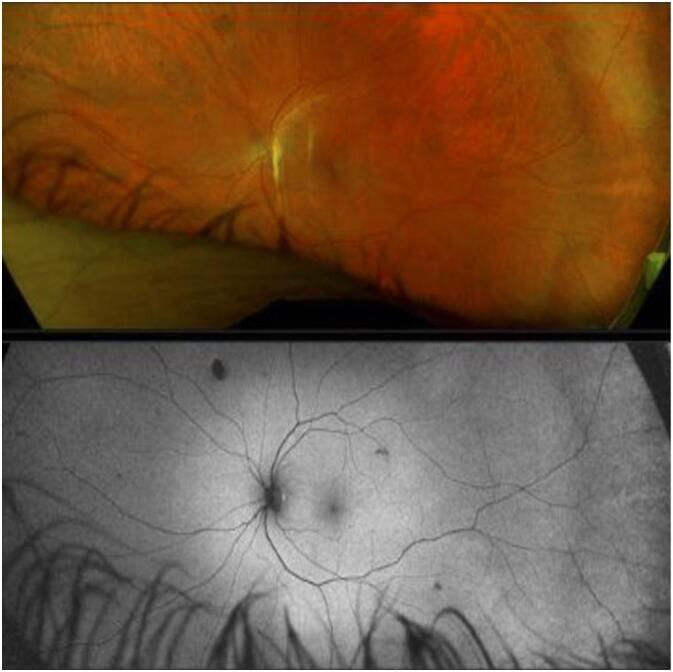
Initial fundoscopic examination OS revealed small CHRPE-like lesions (fewer in number than OD) in multiple retinal quadrants consistent with PO-FLs.

A healthy 42-year-old Caucasian male presented with acute flashes and floaters OS > OD for one week. Best-corrected visual acuity (BCVA) was 20/50 OD and 20/40 OS with normal intraocular pressure OU. External and anterior eye examinations OU were unremarkable. Initial fundoscopic examination OD revealed small to large round CHRPE-like lesions without holes, tears, or macular involvement in multiple retinal quadrants consistent with PO-FLs (oval, fish-tail depigmentation at lesion margin) (Fig. 1). Initial fundoscopic examination OS revealed small CHRPE-like lesions (fewer in number than OD) in multiple retinal quadrants consistent with PO-FLs (Fig. 2). OCT macula imaging showed vitreoretinal attachment without traction. No interventions were performed at this visit. The patient was counseled on CHRPE lesion prognosis, potential changes, and warning signs associated with retinal detachment and tear.

At two-month follow-up, the patient had no acute complaints or reported vision changes. Past medical and surgical history was unchanged from the previous visit. BCVA was 20/30 OD and 20/25 OS; IOP was normal OU. External and anterior eye examinations OU were unremarkable. Fundus eye examination OU showed PO-FLs unchanged from the previous visit, and OCT macula OU showed an unchanged vitreoretinal interface from the last visit. This work has been reported in line with the SCARE criteria [12].

## 3 Discussion

This case study highlights the distinct and crucial ophthalmic findings associated with GS. The constellation of colonic and extra-colonic manifestations observed in GS may aid clinicians in identifying this severe and rare disease associated with significant morbidity and mortality if otherwise undiagnosed [[2], [3], [4], [5],^8^]. CHRPE-like lesions are one such extra-colonic manifestation that may be observed in the general population without an underlying systemic condition. Such lesions (typical CHRPE and bear tracks) have distinct features that suggest benign etiologies rather than potentially severe underlying etiologies [[2], [3], [4],^8^]. It is critical for clinicians to correctly identify and categorize CHRPE-like lesions in order to direct management properly and to avoid misdiagnosis [2,8].

The patient in the present case was diagnosed with GS at age 38 after extra-colonic manifestations (desmoid tumors, osteomas, dental abnormalities, and soft tissue tumors) were observed which prompted a screening colonoscopy that found several adenomatous polyps and was subsequently followed by colon resection. This patient's visits were his first documented eye findings related to GS. PO-FLs typically do not involve the macula and are generally not directly sight-threatening [2,3,8]. These are managed with close ophthalmic observation to identify potential secondary complications, such as vision losses via retinal detachment or tears from nodular growth within PO-FLs [3].

Although rare, these nodules may precipitate vitreomacular traction and central scotoma and can also produce subretinal fluid or exudation which may separate the RPE from the neural retina [8]. There is no documented malignant potential for PO-FLs [5]. Being an early manifestation of GS, PO-FLs (i.e. bilateral, multiple retinal quadrants, irregular and depigmented borders) may prompt clinicians to refer patients for other diagnostic workups such as colonoscopy, sigmoidoscopy, upper endoscopy, ultrasound, computed tomography, magnetic resonance imaging, and/or genetic screening for APC mutations (if ≥10 polyps are present on endoscopy exam) to assess disease manifestations and reduce disease burden [2,4,8].

## Patient consent

Written informed consent was obtained from the patient for publication and any accompanying images. A copy of the written consent is available for review by the Editor-in-Chief of this journal on request.

## Ethical approval

This case report was conducted in accordance with the Declaration of Helsinki and does not require ethical clearance. No surgical or medical interventions were performed for this case, all patient information has been de-identified, and the collection and evaluation of all protected patient health information was performed in a Health Insurance Portability and Accountability Act (HIPAA)-compliant manner.

## Funding

No source of funding.

## Author contribution

**Adrian Babel**: Conceptualization, Methodology, Data curation, Visualization, Validation, Investigation, Writing – Original, Reviewing & Editing.

**Eric K. Chin**: Methodology, Validation, Writing – Reviewing & Editing.

**David RP Almeida**: Conceptualization, Methodology, Data curation, Visualization, Supervision, Validation, Investigation, Writing – Reviewing & Editing.

## Guarantor

David RP Almeida.

Adrian Babel.

## Conflict of interest statement

None.
